# Training guidelines for endovascular stroke intervention: an international multi-society consensus document

**DOI:** 10.1007/s00234-016-1667-0

**Published:** 2016-04-13

**Authors:** S. D. Lavine, K. Cockroft, B. Hoh, N. Bambakidis, A. A. Khalessi, H. Woo, H. Riina, A. Siddiqui, J. A. Hirsch, W. Chong, H. Rice, J. Wenderoth, P. Mitchell, A. Coulthard, T. J. Signh, C. Phatorous, M. Khangure, P. Klurfan, K. ter Brugge, D Iancu, T. Gunnarsson, O. Jansen, M. Muto, I. Szikora, L. Pierot, P. Brouwer, J. Gralla, S. Renowden, T. Andersson, J. Fiehler, F. Turjman, P. White, A. C. Januel, L. Spelle, Z. Kulcsar, R. Chapot, A. Biondi, S. Dima, C. Taschner, M. Szajner, A. Krajina, N. Sakai, Y. Matsumaru, S. Yoshimura, M. Ezura, T. Fujinaka, K. Iihara, A. Ishii, T. Higashi, M. Hirohata, A. Hyodo, Y. Ito, M. Kawanishi, H. Kiyosue, E. Kobayashi, S. Kobayashi, N. Kuwayama, Y. Matsumoto, S. Miyachi, Y. Murayama, I. Nagata, I. Nakahara, S. Nemoto, Y. Niimi, H. Oishi, J. Satomi, T. Satow, K. Sugiu, M. Tanaka, T. Terada, H. Yamagami, O. Diaz, P. Lylyk, M. V. Jayaraman, A. Patsalides, C. D. Gandhi, S. K. Lee, T. Abruzzo, B. Albani, S. A. Ansari, A. S. Arthur, B. W. Baxter, K. R. Bulsara, M. Chen, J. E. Delgado Almandoz, J. F. Fraser, D. V. Heck, S. W. Hetts, M. S. Hussain, R. P. Klucznik, T. M. Leslie-Mawzi, W. J. Mack, R. A. McTaggart, P. M. Meyers, J. Mocco, C. J. Prestigiacomo, G. L. Pride, P. A. Rasmussen, R. M. Starke, P. J. Sunenshine, R. W. Tarr, D. F. Frei, M. Ribo, R. G. Nogueira, O. O. Zaidat, T. Jovin, I. Linfante, D. Yavagal, D. Liebeskind, R. Novakovic, S. Pongpech, G. Rodesch, M. Soderman, A. Taylor, T. Krings, D. Orbach, L. Picard, D. C. Suh, H. Q. Zhang

**Affiliations:** 1 American Association of Neurological Surgeons/ Congress of Neurological Surgeons (AANS/CNS), Rolling Meadows, IL USA; 2 American Society of Neuroradiology (ASNR), Oak Brook, IL USA; 3 Asian Australasian Federation of Interventional and Therapeutic Neuroradiology (AAFITN), Bali, Indonesia; 4 Australian and New Zealand Society of Neuroradiology - Conjoint Committee for Recognition of Training in Interventional Neuroradiology (CCINR) representing the RANZCR (ANZSNR), ANZAN and NSA, Sydney, Australia; 5 Canadian Interventional Neuro Group (CING), Edmonton, AB Canada; 6 World Federation of Interventional and Therapeutic Neuroradiology (WFITN), Gold Coast, Australia; 7 European Society of Neuroradiology (ESNR), Milan, Italy; 8 European Society of Minimally Invasive Neurologic Therapy (ESMINT), Nice, France; 9 Japanese Society for Neuroendovascular therapy (JSNET), Okayama, Japan; 10 Sociedad Ibero Latino Americana de Neuroradiologica (SILAN), Cartagena, Colombia; 11 Society of NeuroInterventional Surgery (SNIS), Washington, DC USA; 12 Society of Vascular and Interventional Neurology (SVIN), Brooklyn, NY USA

## Background

Ischemic stroke is a leading cause of death and disability worldwide. Much of the long-term disability occurs in patients with emergent large vessel occlusion (ELVO). In fact, in these patients, occlusion of a major intracerebral artery results in a large area of brain injury often resulting in death or severe disability [1]. Until recently, intravenous tissue plasminogen activator (t-PA) was the only proven treatment for ELVO.

However, the landscape of stroke treatment has changed with the publication of five randomized multicenter controlled clinical trials. These trials provide class 1, level A evidence that endovascular thrombectomy (ET) is the standard of care for patients with ELVO. In particular, thrombectomy results in significantly better clinical outcomes compared to best medical therapy in patients with acute occlusion of the intracranial internal carotid artery (ICA) and/or M1 segment of the middle cerebral artery (MCA) [2–6]. These results have led to guideline recommendations advocating for endovascular treatment in addition to t-PA for patients with ELVO. In addition, ET is now offered as first-line therapy for patients that are not eligible for intravenous thrombolysis [7–9]. However, achieving the best possible clinical outcomes with endovascular stroke treatment mandates structured training and education of those physicians who are providing endovascular stroke treatment. On this regard, a recent meta-analysis of these five clinical trials showed that the vast majority of thrombectomies were performed by experienced neurointerventionalists. These include interventional neuroradiologists, endovascular neurosurgeons, and interventional neurologists who routinely perform neuroendovascular procedures [10]. None of the studies allowed physicians without previous experience in mechanical thrombectomy to enroll patients. The centers participating in these trials offered endovascular stroke therapy 24 h a day (with the exception of those in the EXTEND-IA trial) with expertise in vascular neurology and neurocritical care in a comprehensive stroke center. On-site expertise in vascular neurology and neurocritical care is paramount to achieving good clinical outcomes.

Geographical limitations to rapid access to acute stroke centers providing mechanical thrombectomy have led some to suggest physicians without prior experience or formal neuroendovascular training should consider providing coverage for these procedures. A multidisciplinary British Intercollegiate Stroke Working Party put forth a document outlining the safe delivery of mechanical thrombectomy, which highlights that operators should not normally carry out procedures with which they are unfamiliar and that they should recognize ad-hoc arrangements are not in the best interest of patients.

It is also important to recognize that modern endovascular stroke therapy focuses on direct clot removal with mechanical devices, as compared with previous paradigms where intra-arterial thrombolytic infusion was an acceptable treatment option for large vessel occlusions [11]. The technical skills needed to safely deliver devices into the intracranial circulation are significantly more involved than simply placing a catheter for medication infusion. Catheter skills from other circulations do not replace the need for formal training in safe intracranial microcatheter navigation and device placement.

Acute ischemic stroke is a complex disease and successful endovascular treatment is based on the comprehensive ability to rapidly integrate multiple pieces of information, including: the patient’s history, clinical examination, neuroradiological studies, and to subsequently formulate a treatment plan. Both patient selection and procedural expertise are critical to achieve a good clinical outcome. Hence, there is a clear rationale for formal training in both clinical neuroscience and interventional neuroradiology.

The purpose of this document is to define what constitutes adequate training for physicians who can provide endovascular treatment for acute ischemic stroke patients. These training guidelines are modeled after prior standards of training documents such as the training, competency, and credentialing standards for diagnostic cerebral angiography, carotid stenting and cerebrovascular intervention [12], and the performance and training standards for endovascular ischemic stroke treatment [13], written and endorsed by multispecialty groups. In addition, the importance of organ specific training, rigorous quality improvement benchmarks, and minimum volume requirements needed to maintain high quality care has been extensively described for acute myocardial infarction, an analogous time sensitive disease [14].

This document represents the cumulative work of the societies listed below and represents an international consensus on adequate training to safely and effectively perform these procedures:

- American Academy of Neurological Surgeons/Congress of Neurological Surgeons (AANS/CNS)
- American Society of Neuroradiology (ASNR)
- Asian Australasian Federation of Interventional and Therapeutic Neuroradiology (AAFITN)
- Australian and New Zealand Society of Neuroradiology—Conjoint Committee for Recognition of Training in Interventional Neuroradiology (CCINR) representing the RANZCR (ANZSNR), ANZAN and NSA
- Canadian Interventional Neuro Group (CING)
- European Society of Neuroradiology (ESNR)
- European Society of Minimally Invasive Neurologic Therapy (ESMINT)
- Japanese Society for Neuroendovascular therapy (JSNET)
- Sociedad Ibero Latino Americana de Neuroradiologica (SILAN)
- Society of NeuroInterventional Surgery (SNIS)
- Society of Vascular and Interventional Neurology (SVIN)
- World Federation of Interventional and Therapeutic Neuroradiology (WFITN)

## Physician qualifications

Physicians providing intra-arterial treatment for acute stroke are required to have appropriate training and experience for the performance of neuroangiography and interventional neuroradiology.

We recognize that the specific training pathways may differ across nations, but the consensus is to mandate adequate training to perform emergent endovascular stroke intervention. These cognitive requirements consist of baseline training and qualifications as well as ongoing professional education, which are essential for safe and efficient patient management.

It is also important to point out that these qualifications are for new practitioners who are not currently performing acute stroke intervention with mechanical thrombectomy. We understand that there are current practitioners (who are board certified or board eligible in radiology, neurology or neurosurgery) who may have trained prior to the establishment of formal training pathways and have acquired the necessary skills listed below to safely and effectively treat these complex patients. We would still expect the same requirements for maintenance of qualifications as listed below.

### Baseline training and qualifications:

1. Residency training (in radiology, neurology or neurosurgery) which should include documented training in the diagnosis and management of acute stroke, the interpretation of cerebral arteriography and neuroimaging under the supervision of a board-certified neuroradiologist, neurologist or neurosurgeon with subsequent board eligibility or certification. The residency program and supervising physicians should be accredited according to national standards as they pertain to the countries involved. Those physicians who did not have adequate such training during their residencies must spend an additional period (typically one year) by training in clinical neurosciences and neuroimaging, focusing on the diagnosis and management of acute stroke, the interpretation of cerebral arteriography and neuroimaging prior to their fellowship in neuroendovascular interventions.
  AND
2. Dedicated training in Interventional Neuroradiology (also termed Endovascular Neurosurgery or Interventional Neurology) under the direction of a Neurointerventionalist (with neuroradiology, neurology or neurosurgical training background), at a high-volume center. It is preferred that this is a dedicated year, which occurs after graduating from residency (i.e., a fellowship). A training program accredited by a national accrediting body is also strongly preferred but not required. Published standards exist for various countries [15–21]. Within these programs, specific training for intra-arterial therapy for acute ischemic stroke should be performed, including obtaining appropriate access even in challenging anatomy, microcatheter navigation in the cerebral circulation, knowledge, and training of the use of stroke specific devices and complication avoidance and management.

While various national standards will have differing procedure requirements, we encourage practitioners to meet their national minimum procedural and training standards. Fellowships which are not accredited by national credentialing bodies should still have adequate training to meet their local minimum procedure requirements. In addition, we expect that minimum training numbers for stroke thrombectomy may increase in future revisions of these standards given the recent developments in the field.

### Maintenance of physician qualifications:

It is vital that the physician has ongoing stroke-specific continuing medical education. A minimum of 16 h of stroke specific education every 2 years is suggested. Individual physician outcomes should conform to national standards and institutional requirements. In addition, the physician should participate in an ongoing quality assurance and improvement program. The goals of this quality assurance program for stroke therapy would be to monitor outcomes both in the peri-procedural period and at 90 days. The quality assurance program must review all emergency interventional stroke therapy patients. In addition, participation in a national quality improvement registry, when available, is also encouraged. Outcomes should be tracked and recorded. While threshold levels for recanalization, complication rates, etc. have yet to be established, we suggest the following as a minimum:

1. Successful recanalization (modified TICI 2b or 3) in at least 60 % of cases.
2. Embolization to new territory of less than 15 %.
3. Symptomatic intracranial hemorrhage (i.e., parenchymal hematoma on imaging with clinical deterioration) rate less than 10 %.

### Hospital requirements:

Successful treatment of the ELVO patient does not occur in a vacuum, but rather with the framework of a multi-disciplinary team. As such, we feel it is critical that the patients be treated in a center, which has 24/7 access to the following:

1. Angiography suites suitably equipped to handle these patients, as well as equipment and capability to handle the complications.
2. Dedicated stroke and intensive care units (preferably dedicated neuro-intensive care unit), staffed by physicians with specific training in those fields.
3. Vascular neurology and Neurocritical care expertise.
4. Neurosurgery expertise, including vascular neurosurgery.
5. All relevant neuroimaging modalities (CT/CTA, MR/MRA, Trans-cranial Doppler [TCD]), including 24/7 access to CT and MRI.

## Summary

We, as a group of international multi-disciplinary neurointerventional societies involved in the endovascular management of acute ischemic stroke, have put forth these training guidelines. We believe that a neuroscience background, dedicated neurointerventional training, and stringent peer review and quality assurance processes are critical to ensuring the best possible patient outcomes. Well-trained neurointerventionalists are a critical component of an organized and efficient team needed to deliver clinically effective mechanical thrombectomy for acute ischemic stroke patients.
